# Mental Health Challenges during COVID-19 Pandemic: Experiences of Primary Healthcare Nurses in Durban, South Africa

**DOI:** 10.3390/ijerph20176683

**Published:** 2023-08-30

**Authors:** Stanley Chibuzor Onwubu, Maureen Nokuthula Sibiya, Mokgadi Ursula. Makgobole

**Affiliations:** 1 Department of Chemistry, Durban University of Technology, Durban 4001, South Africa; 2 Division of Research, Innovation and Engagement, Mangosuthu University of Technology, Umlazi 4031, South Africa; sibiya.nokuthula@mut.ac.za; 3 Department of Somatology, Durban University of Technology, Durban 4001, South Africa; mokgadim@dut.ac.za

**Keywords:** COVID-19, mental healthcare, primary healthcare nurses, sub-Saharan Africa, challenges, adaptation, outreach, telehealth, vaccine hesitancy, mental health awareness

## Abstract

The COVID-19 pandemic had a significant impact on the mental health of individuals globally, and primary healthcare (PHC) nurses play a critical role in providing mental healthcare services. However, limited research has explored the experiences of PHC nurses in providing mental healthcare services during the COVID-19 pandemic. This study explored the experiences of PHC nurses in providing mental healthcare services during the pandemic in Durban, South Africa. The aim was to identify the challenges faced by healthcare providers and the potential for innovative approaches to improve access to care. A qualitative, exploratory design guided the study, and data were collected through in-depth interviews with twelve PHC nurses purposively selected. Thematic analysis was used to analyze the data. Findings from interviews with PHC nurses reveal that the pandemic exacerbated existing challenges, including medication adherence issues, fear and uncertainty among patients, vaccine hesitancy, decreased clinic visits, and the mental and emotional toll on both patients and healthcare workers. PHC nurses adapted their services by increasing outreach efforts, prioritizing patient care, and utilizing technology and non-governmental organizations’ (NGOs) support. Challenges included reduced patient visits, complexities in healthcare provision, and a lack of adequate support. Positive changes observed include increased mental health awareness among healthcare professionals and younger generations. Recommendations include implementing outreach and awareness campaigns, providing accurate information about COVID-19 and vaccinations, and promoting cultural sensitivity in mental healthcare provision.

## 1. Introduction

Mental health is an essential component of overall health and well-being. However, mental health services in many low- and middle-income countries (LMIC), particularly in Sub-Saharan Africa (SSA), are often limited [1,2]. In South Africa, mental health promotion, prevention of mental disorders, and provision of mental health care are basic services that are provided in primary health care (PHC) clinics [3]. About 23% of people attending PHC clinics suffer from mental health disorders [4]. Despite the high number of people with mental health conditions, mental health has a low priority in South Africa, and people with mental health disorders do not receive the care they require in PHC clinics [4]. People with mental illness often experience greater difficulty accessing mental health services due to different attitudinal and structural barriers [5,6]. To narrow the treatment gap, many countries, including South Africa, adopted the initiative of integrating mental health services into primary healthcare [7,8]. The Policy on Integration of Mental Health Care into PHC in South Africa was promulgated in 1997. According to this policy, mental health care users should receive treatment at the clinic near where they live, and mental health care services must be integrated into general care at PHC [9]. Despite efforts to increase the accessibility of mental health services, treatment rates for mental illness remain low in LMIC. The COVID-19 pandemic had a significant impact on mental health globally, with reports of increased anxiety, depression, and other mental health problems [10,11,12]. The pandemic disrupted many aspects of life, including economic activities, education, and social interactions [13,14]. These disruptions resulted in increased stress, anxiety, and depression, which contributed to the overall increase in mental health disorders [14,15,16].

SSA is a region that is particularly vulnerable to the mental health impacts of the COVID-19 pandemic [17]. The region has a high burden of mental health disorders, with an estimated 13.5% of the population experiencing a mental health disorder at any given time [18]. According to the 2019 global burden of disease estimate, more than 970 million people live with mental illness, which indicates a 48.1% increase since 1990. Of all people with mental illness, 66.7% reside in low and middle Sustainable Development Index (SDI) countries [19]. Furthermore, the pandemic has had a significant impact on the mental health of individuals on the continent, with reports of increased anxiety, depression, and other mental health problems [17,20]. Due to the COVID-19 pandemic, mental illness has posed a great concern, with a 25% worldwide increase in the prevalence of depression and anxiety [2]. South Africa is no exception, as it was severely impacted by the COVID-19 pandemic, with one of the highest numbers of reported cases and deaths on the African continent [21].

Studies have reported that major and sustained disease outbreaks such as COVID-19 have caused physical, mental, and psychological distress in society as a whole and may be attributed to an increased risk for depression, anxiety, panic attacks, somatic symptoms, posttraumatic stress disorder (PTSD), psychosis, suicidal ideation, and a low quality of life [22,23]. PHC nurses play a critical role in providing mental healthcare services in South Africa [24]. However, South Africa, like other countries in SSA, faces significant challenges in providing adequate mental healthcare services, including a shortage of mental healthcare workers, limited access to mental healthcare services, and stigma associated with mental health problems [17,20,25]. The COVID-19 pandemic has further exacerbated these challenges, with PHC nurses facing increased workload and limited access to personal protective equipment (PPE) while providing mental healthcare services [26]. Prioritizing the physical health of society with little attention to the mental strain that the pandemic has caused will likely increase the risk of experiencing another ‘pandemic’ linked to the development of mental disorders [27].

While PHC nurses play a critical role in providing mental healthcare services in SSA, little is known about their experiences during the pandemic in Durban, South Africa. The purpose of the study was to explore the experiences of PHC nurses in providing mental healthcare services during the COVID-19 pandemic in Durban, South Africa. The study aimed to explore the experiences of PHC nurses in providing mental healthcare services during the pandemic in Durban, South Africa, as well as the potential for innovative approaches to improving access to care. The study findings thus contribute to the broader effort to improve the provision of mental healthcare services during the COVID-19 pandemic in SSA.

## 2. Materials and Methods

### 2.1. Research Design

The study used a qualitative, exploratory design, which is appropriate for gathering rich, in-depth data on the experiences and perspectives of PHC nurses. According to Blumberg, et al. [28], the qualitative research approach follows the interpretivist research paradigm, which is premised on how people make sense of the world around them by sharing their experiences with others through the medium of language.

### 2.2. Sampling and Participants

The study used purposive sampling to identify participants who are PHC nurses providing mental healthcare services in Durban, South Africa. Twelve PHC nurses with vast experience in mental healthcare were recruited from public and private healthcare facilities, including clinics and hospitals. The inclusion criteria include PHC nurses actively working in health care facilities in Durban, South Africa, PHC nurses that have direct contact and are involved in providing healthcare services during the COVID-19 pandemic, PHC nurses with a minimum of one year of experience working in a primary healthcare setting, and PHC nurses who are proficient in English, the language in which the study will be conducted and reported. The exclusion criteria include individuals who are not primary healthcare nurses, such as administrative staff or non-clinical employees, PHC nurses who do not have direct patient contact or who are not involved in providing healthcare services during the pandemic, PHC nurses with less than one year of experience working in primary healthcare settings, PHC nurses who are not proficient in English, the language in which the study will be conducted and reported, and nurses who are retired and no longer actively working in healthcare facilities.

### 2.3. Data Collection

The Data for the study were collected through semi-structured interviews with PHC nurses between April and May 2023. The interviews were conducted in person and were audio-recorded with the participant’s permission. The interview guide was developed based on research questions and sub-questions, and the pilot was tested with a small group of participants (n = 3) at the Isolempilo Clinic in Durban to ensure clarity and relevance. To establish the views of PHC nurses, the following main questions were asked:

What are the challenges faced by PHC nurses in providing mental healthcare services and the innovative approaches used to address these challenges during the COVID-19 pandemic in Durban, South Africa?

What changes have PHC nurses observed in the mental health status of their patients during the COVID-19 pandemic in Durban, South Africa?

What are the barriers to providing mental healthcare services during the COVID-19 pandemic in Durban, South Africa?

What are the facilitators to providing mental healthcare services during the COVID-19 pandemic in Durban, South Africa?

What strategies have PHC nurses used to adapt their mental healthcare services to the COVID-19 pandemic in Durban, South Africa?

What are the perceptions of PHC nurses regarding the effectiveness of their mental healthcare services during the COVID-19 pandemic in Durban, South Africa?

### 2.4. Data Analysis

The audio recordings of the interviews were transcribed verbatim and analyzed using thematic analysis with the aid of NVivo software. This approach involves coding the data to identify patterns, themes, and categories that emerge from the data [29]. The analysis was conducted iteratively, with the researchers reviewing the data to refine and revise the themes and categories until a coherent and comprehensive understanding of the data was achieved.

### 2.5. Trustworthiness and Credibility

The trustworthiness and credibility of the study were ensured through a rigorous and transparent research design, data collection, and data analysis process. The accuracy of the transcription was ensured by listening to the recordings again before data analysis. Before data transcription and analysis, a debriefing session with the participants was performed. Each of the participants was asked to give a summary of his or her dialogue and discussion.

### 2.6. Ethical Considerations

The study was conducted in accordance with ethical principles, including obtaining informed consent from participants, protecting participant confidentiality, and ensuring that the research does not cause harm to participants or others. The study was approved by the relevant institutional review board(s) before data collection began (RDI/06/2023).

## 3. Results

Table 1 highlights the themes and subthemes that were extracted from the study’s findings. These themes and subthemes are discussed next.

### 3.1. Theme 1: Impact of COVID-19 on Mental Healthcare Provision

Five sub-themes have been highlighted under the impact of COVID-19 on mental healthcare provision, which are medical adherence challenges, fear and uncertainty, reluctance to vaccination, decrease in clinic visits, and mental and emotional toll.

#### 3.1.1. Medication Adherence Challenges

Participant 1 observed that medication adherence was already a challenge, but the COVID-19 pandemic made it worse. Many patients could not come to the clinic due to transportation issues caused by lockdowns and restrictions. This resulted in difficulties for patients to access their chronic medications, leading to a decline in medication adherence rates during the pandemic.

*“What I noticed was I think adherence to medication, we are struggling with patients adhering to medication already, but I think it made it worse in terms of people who couldn’t come to the clinic because of the transportation.”*

[Participant 1]

#### 3.1.2. Fear and Uncertainty

Participant 3 highlighted that the COVID-19 pandemic had a significant impact on people’s mental health due to fear and uncertainty. The fear of the unknown and witnessing the high number of deaths caused distress and mental disturbance among many individuals. The lack of understanding about the virus and its consequences added to the anxiety and uncertainty experienced by people during the pandemic.

*“I think it had a great impact because people were scared, they didn’t know what it is, and a lot of people were dying. So, I think a lot of people got disturbed mentally as well because they didn’t really know what to expect.”*

[Participant 3]

#### 3.1.3. Reluctance to Vaccination

Participant 4 mentioned that there was a noticeable reluctance among some individuals to take the COVID-19 vaccines. This hesitancy was attributed to reports of adverse effects experienced by some vaccinated individuals, leading others to fear potential negative outcomes. As a result, vaccine hesitancy affected the willingness of certain people to get vaccinated during the pandemic.

*“They are now very reluctant to take vaccines. Because some of them they experience sort of like more of the illnesses when they took it than when they were not, so they didn’t do good, and it did worse instead of good.”*

[Participant 4]

#### 3.1.4. Decrease in Clinic Visits

Participant 4 noted that there was a significant decrease in the number of patients visiting the clinic during the COVID-19 pandemic. Many people were afraid to come to healthcare facilities due to the fear of exposure to the virus. As a result, the clinic experienced a decline in patient attendance, which affected the delivery of healthcare services during the pandemic.

*“Yeah, there were. They were even afraid to come to the facility, to the polls, that’s where they were meeting more people, than when they were. So, they felt safer. So, we experience like a drop of people coming to the clinic.”*

[Participant 4]

#### 3.1.5. Mental and Emotional Toll

Participant 2 expressed that the impact of the COVID-19 pandemic on patients’ mental health was profound. The phrase “they were all flat” suggests a sense of emotional and psychological heaviness or lack of energy among the patients. Furthermore, Participant 2 indicated that the pandemic had a widespread effect on both patients and healthcare workers, indicating the significant toll it took on the mental well-being of individuals during this challenging time.

*“They were all flat. We were all affected.”*

[Participant 2]

### 3.2. Theme 2: Adaptation of Mental Healthcare Services

Three key strategies for mental healthcare services were uncovered from the interview discussion, namely increased outreach and support, prioritizing patient care, and the use of digital technologies and intervention from non-governmental organizations (NGOs).

#### 3.2.1. Increased Outreach and Support

Participant 1 mentioned that during the COVID-19 pandemic, there was an increased effort to provide outreach and support to patients. Healthcare workers took the initiative to reach out to patients, allaying their anxiety and offering reassurance. The focus shifted from patients having to come to the clinic to receive care to healthcare workers proactively reaching out to provide the needed support, including medication assistance and emotional reassurance. This adaptation aimed to address the challenges posed by restrictions and transportation issues during the pandemic.

*“Just by allaying anxiety to patients assuring them, and us as healthcare workers reaching out to them more than them coming to us for their medication.”*

[Participant 1]

#### 3.2.2. Prioritizing Patients’ Care

Participant 5 highlighted the importance of prioritizing patients’ care during the COVID-19 pandemic. The focus was on fast-tracking the treatment of patients with major cases and chronic conditions to avoid any potential security burden or complications. By ensuring timely and prioritized care for patients, healthcare workers aimed to address their needs promptly and effectively, despite the challenges posed by the pandemic.

*“The major cases are obviously those on treatment. Don’t let them wait too long time because they can be a security burden and can trigger anything with them, so try to fast-track and make them priority.”*

[Participant 5]

#### 3.2.3. Use of Technology and NGOs

Participant 5 mentioned the use of technology and NGOs to provide mental healthcare services during the COVID-19 pandemic. They utilized digital means to locate patients and offer support, addressing their fears and needs promptly. This adaptation involved utilizing technology in mental healthcare, which might not have been extensively used before. Additionally, NGOs played a role in providing staffing and support, extending their services to include mental healthcare during the pandemic.

*“Yeah, we do. We do have our digital dreams that go out here and try and locate patients. They don’t come on time and try to address their fears and really help them by taking whatever they need to their and things like that. So, we did try and adapt using NGOs as our staffing.”*

[Participant 5]

While the technology and NGO involvement were not entirely new, they were modified to cater to mental healthcare needs, especially during the challenging circumstances of the pandemic.

*“It was always there, but not so much mental health uses, more with chronic uses and CD’s, so we try to talk with everybody.”*

[Participant 5]

Participant 1 acknowledged the significant role of technology, particularly social media and the internet, in delivering mental healthcare services during the COVID-19 pandemic. The accessibility of information through digital platforms allowed healthcare providers to stay updated and informed about the latest developments, guidelines, and mental health resources. These digital tools facilitated the dissemination of mental health-related information, created awareness, and provided support to both healthcare workers and the general public during the challenging times of the pandemic.

*“Yeah, I think it did play a role because mostly we were being updated by the social media, the Internet yeah, I think it is.”*

[Participant 1]

### 3.3. Theme 3: Challenges in Providing Mental Healthcare Services

Three key challenges were uncovered from the interview discussion, namely a reduced number of patients, the complexity of healthcare provision during the pandemic, and a lack of adequate support.

#### 3.3.1. Reduced Number of Patients

Participant 1 pointed out that one of the challenges in providing mental healthcare services during the pandemic was the reduced number of patients visiting the clinic. The lack of patients limited the opportunity for healthcare providers to teach and educate about mental health, as they would typically do. This decrease in patient visits impacted the delivery of mental health education and support.

*“Challenges, I think there were challenges because we couldn’t get people coming into the clinic. So, we couldn’t teach about mental health as we would because there were no patients coming in.”*

[Participant 1]

#### 3.3.2. Complexity of Healthcare Provision during the Pandemic

Participant 2 mentioned the challenges faced in providing mental healthcare services while simultaneously dealing with the demands of COVID-19 testing. The clinic was running out of testing kits at times, leading to stress and pressure on healthcare workers. Juggling both mental health counseling and COVID-19 testing added to the complexity of healthcare provision during the pandemic.

*“They want to challenge you. We had to counsel the patient and inspect the patient every morning, that could help to relieve the stress It will meet. The challenges were when we were running out of these kits because we were doing COVID-19 testing in our site, so sometimes we do run out of stock and right.”*

[Participant 2]

#### 3.3.3. Lack of Adequate Support

Participant 5 shared a coping mechanism during the COVID-19 pandemic, emphasizing the significance of support from family. They found strength in the support they received from their family members on a daily basis, which helped them cope with the challenges of their work as a healthcare provider during the pandemic. The family’s support acted as a source of motivation and resilience, enabling Participant 5 to continue providing care and support to patients despite facing difficulties during this time.

*“Well, the support from your family comes most, so you know every day while you’re coming to work and when you’re doing it, it makes you stronger.”*

[Participant 5]

### 3.4. Theme 4: Positive Changes from Providing Mental Healthcare Services during the Pandemic

Two positive changes from providing mental healthcare services during the pandemic were uncovered from the interview discussion, namely awareness of mental health and community acceptance of mental health.

#### 3.4.1. Awareness of Mental Health

Participant 1 mentioned that a positive change observed during the pandemic was an increased awareness of mental health among healthcare professionals like themselves. They recognized the importance of mental healthcare and were educated on the subject. However, in their community, the general awareness about mental health was still limited, with only educated individuals and teenagers being more informed about it due to mental health education at schools. This highlights the need for further efforts to raise awareness about mental health among the broader population, including the elderly and less educated individuals.

*“Yeah, I think, but as I am saying in my community as much as we are aware of it be-cause we’re professional nurses, but the community is not so much aware about the mental health situation. It’s only the people that are educated and mostly the teenagers that are still in school because it is taught at schools, but not the elderly people.”*

[Participant 1]

#### 3.4.2. Community Acceptance of Mental Health

Participant 1 shared insights into perceptions of mental health in their community, particularly in rural areas. They mentioned that in such communities, mental health is sometimes perceived through a traditional lens and viewed differently based on the level of education. This highlights the importance of cultural and educational factors in shaping people’s attitudes toward mental health. There may be varying levels of acceptance and awareness of mental health issues, influenced by cultural beliefs and educational backgrounds. It indicates the need for targeted awareness and education programs to address misconceptions and improve mental health acceptance in such communities.

*“Some of them because in the community that I live in. I live in the rural areas; so, sometimes mental health is perceived as traditional. It is viewed differently; it depends on the level of education.”*

[Participant 1]

Participant 1 mentioned that in their community, mental health awareness is higher among educated individuals and mostly teenagers who receive education about mental health at school. However, there seems to be a lack of awareness about mental health among the elderly and the broader community. This observation highlights the potential positive change of mental health education being integrated into schools, enabling younger generations to be more aware and informed about mental health issues. However, there is still a need to improve mental health awareness among other segments of the community, particularly the elderly population, to promote better mental healthcare provision during the pandemic and beyond.

*“Yeah, I think, but as I am saying in my community as much as we are aware of it be-cause we’re professional nurses, but the community is not so much aware about the mental health situation. It’s only the people that are educated and mostly the teenagers that are still in school because it is taught at schools, but not the elderly people.”*

[Participant 1]

### 3.5. Theme 5: Suggestions for Improving Mental Healthcare Services

Two suggestions were offered by the PHC nurses on how to improve mental healthcare services. This includes outreach and awareness campaigns and providing accurate information.

#### 3.5.1. Outreach and Awareness Campaign

Participant 3 suggested that improving mental healthcare services could be achieved through increased outreach efforts and awareness campaigns. They emphasized the importance of talking openly about mental health conditions to make people aware that it is okay to have such issues and that life can continue positively despite facing mental health challenges. The goal is to reduce stigma and promote acceptance, fostering a supportive environment for individuals experiencing mental health issues.

*“Having more outreach and talking about the mental health condition and making people aware of them and that they are ok, and life does continue even though you do have the mental issue.”*

[Participant 3]

#### 3.5.2. Providing Accurate Information

Participant 4 highlighted the importance of providing accurate information to the public about COVID-19 and vaccinations. They expressed concern that insufficient information led to misconceptions and fear about vaccines, resulting in a reluctance to get vaccinated. Participant 4 stressed the need to educate individuals about the virus, vaccine benefits, and potential side effects, allowing them to make informed decisions. By offering comprehensive information, healthcare providers can dispel myths and build trust, encouraging more people to get vaccinated and promoting public health during the pandemic.

*“Giving them the vaccine than giving them the information. But the virus is all about the dangers and all what to expect, so people were ending up using what happened to somebody taking as what exactly is happening then experimenting like things on their own. Like if somebody passed on, they say the vaccine has caused somebody to die. But if we can give more information to people so that they can have the choice, more informed and make decisions.”*

[Participant 4]

## 4. Discussion

The COVID-19 pandemic has had significant impacts on mental healthcare services in SSA, where mental healthcare services are already under-resourced. The purpose of the study is to explore the experiences of PHC nurses in providing mental healthcare services during the COVID-19 pandemic in Durban, South Africa. The study aims to identify the challenges faced by PHC nurses in providing mental healthcare services during the pandemic, as well as the potential for innovative approaches to improving access to care.

### 4.1. Impact of COVID-19 on Mental Healthcare Provision

The findings of the study suggest that the impact of the COVID-19 pandemic on mental healthcare provision is multifaceted. It was found that the COVID-19 pandemic exacerbated existing challenges related to medication adherence. With transportation issues caused by lockdowns and restrictions, many patients could not access their chronic medications, leading to a decline in adherence rates. This can have serious consequences for patients with chronic mental health conditions, as interrupted or inconsistent medication use may result in symptom exacerbation and a higher risk of relapse [30]. From a practical perspective, healthcare providers need to find alternative ways to support patients’ medication adherence during crises or periods of restricted mobility. Telemedicine and home delivery services for medications could be viable solutions to address this issue [31]. Moreover, the finding highlights the importance of providing patients with sufficient medication supplies to sustain them through emergencies.

Equally, the fear of the unknown and the high mortality rates caused distress and mental disturbance among individuals during the pandemic. It was uncovered that the lack of understanding about the virus and its consequences added to the anxiety and uncertainty experienced by people. Given this concern, mental health services should prioritize providing support and resources to help individuals cope with fear and uncertainty during pandemics [32]. Psychoeducation about the virus and its transmission, as well as information on coping strategies, can be valuable in reducing anxiety [33]. Thus, timely dissemination of accurate information and addressing misconceptions are crucial in mitigating the mental health impact of fear and uncertainty.

Furthermore, it occurred that vaccine hesitancy among certain individuals was a challenge during the COVID-19 pandemic. It was found that the reports of adverse effects experienced by some vaccinated individuals contributed to vaccine hesitancy. Addressing vaccine hesitancy requires a multi-faceted approach involving healthcare providers, community leaders, and public health authorities. Transparent communication about vaccine safety, effectiveness, and benefits is essential to building trust and increasing vaccination rates [34]. In this regard, mental health professionals can play a role in addressing vaccine-related anxieties and helping individuals make informed decisions [35].

The fear of exposure to the virus led to a significant decrease in clinic visits during the pandemic. It was uncovered that reduced patient attendance impacted the delivery of healthcare services. Given this concern, telehealth and virtual care options should be expanded and promoted to ensure continuous access to mental healthcare during crises. Telehealth services have been found effective in providing mental health support during the pandemic, enabling remote consultations, and reducing exposure risks [36]. To achieve this, healthcare providers should implement safety measures and communicate them effectively to alleviate patient concerns about visiting healthcare facilities [37].

Moreso, it was found that the pandemic had a profound impact on patients’ mental health, as well as healthcare workers. Both groups experienced emotional and psychological challenges during this time. Healthcare workers’ mental health should be a priority, as they play a crucial role in providing care and support to patients [24]. Adequate support systems, such as counseling services and mental health check-ins, should be implemented for healthcare professionals [38]. Additionally, integrating mental health support into overall healthcare services can help address the emotional toll experienced by patients during pandemics [32].

### 4.2. Adaptation of Mental Healthcare Services

During the pandemic, healthcare workers took a proactive approach by reaching out to patients to offer support and reassurance. This shift in focus from patients coming to the clinic to healthcare workers reaching out aimed to address challenges such as restrictions and transportation issues. The finding suggests that the increased outreach and patient support approach should be integrated into regular mental healthcare practices beyond the pandemic. Telemedicine and remote consultations can be incorporated to maintain continuous communication with patients [39]. Regular check-ins and proactive mental health support can help enhance patient engagement and adherence to treatment plans.

Also, recognizing the importance of timely care, it was found that healthcare workers prioritized patients with major cases and chronic conditions to avoid potential complications. This was achieved by fast-tracking treatments aimed at addressing patients’ needs promptly despite the challenges posed by the pandemic. Hence, prioritizing patients’ care should remain a fundamental principle in mental healthcare provision during emergencies or crisis situations [40]. To achieve this, healthcare systems need to develop protocols to ensure efficient triaging and prompt care for patients with severe mental health conditions [41]. Clear guidelines and collaboration between mental health professionals and other healthcare providers can help streamline patient care during challenging times. This is particularly important in light of the report by Kohn, et al. [42] that collaboration and information flows between healthcare professionals and persons with severe mental illness are troublesome. According to the authors, modifying these aspects will improve the quality of somatic healthcare for vulnerable patients [42].

Of interest, the pandemic led to the increased use of technology to provide mental health services. Digital means, including social media and digital platforms, facilitate communication, patient location, and information dissemination. NGOs also played a role in providing staffing and support for mental healthcare services during the pandemic. The integration of technology and NGO support in mental healthcare delivery should be continued and expanded upon. Healthcare systems can invest in telehealth infrastructure and training for mental health professionals to utilize technology effectively in service delivery [43]. Additionally, collaborating with NGOs and community-based organizations can help extend mental health services to underserved populations, even during normal times. This is consistent with Ojagbemi and Gureje [44], who suggested a collaboration between faith-based mental healthcare and health practitioners may help bridge the large treatment gap for mental health conditions on the African continent.

### 4.3. The Challenges Faced in Providing Mental Healthcare Services during the COVID-19 Pandemic

Equally relevant, it was found that the challenges in providing mental healthcare services during the COVID-19 pandemic call for a multifaceted approach. For instance, it was uncovered that the reduced number of patients visiting the clinic during the pandemic posed a challenge in delivering mental health education and support. With fewer patients, healthcare providers had limited opportunities to engage with individuals and raise awareness about mental health. It is therefore necessary that healthcare systems explore alternative ways to reach out to individuals and provide mental health education and support beyond clinic visits. Telehealth platforms and digital outreach can be effective in disseminating mental health information to a broader audience [45,46]. Additionally, community-based mental health campaigns and public awareness initiatives can play a vital role in promoting mental health education [47].

Furthermore, it was uncovered that the COVID-19 pandemic brought the challenge of juggling mental healthcare services with the demands of COVID-19 testing. It was found that healthcare workers faced stress and pressure while managing both aspects of care provision. During public health crises, it is advisable that healthcare systems ensure adequate resources and support to manage the complexities of multiple healthcare services [48]. This may involve enhancing staffing, training, and logistics to handle the dual responsibilities efficiently. Equally important, flexibility in adapting healthcare workflows during pandemics can help healthcare providers maintain quality mental healthcare alongside other essential services.

### 4.4. Positive Changes from Providing Mental Healthcare Services during the Pandemic

From the study’s findings, there were some positive changes observed during the pandemic in the context of mental healthcare provision. It was found that healthcare professionals experienced a positive change in increased awareness of mental health during the pandemic. They recognized the importance of mental healthcare and were educated on the subject. However, some of the healthcare practitioners point out that the general awareness about mental health in the broader community, particularly among the elderly and less educated individuals, is still limited. Efforts to raise awareness about mental health should be prioritized and extended to reach all segments of the community, especially those with limited access to mental health education. Community-based awareness campaigns, outreach programs, and public health initiatives can play a vital role in improving mental health literacy and promoting destigmatization [49]. Additionally, integrating mental health education into schools can continue to be beneficial in fostering a mentally informed and aware younger generation [50].

The influence of cultural and educational factors in shaping perceptions of mental health in their community, particularly in rural areas, was also highlighted. Mental health was sometimes perceived through a traditional lens, with varying levels of acceptance based on educational backgrounds. According to Moleiro, et al. [51], cultural competence and sensitivity are crucial when providing mental healthcare services in diverse communities. Thus, mental health programs should be tailored to address specific cultural beliefs and values to improve acceptance and engagement with mental health services [52]. Given the significance of culture, one could rightly infer that collaborating with community leaders and involving community members in mental health initiatives can help bridge cultural gaps and ensure that services are responsive to the needs of the population.

### 4.5. Suggestions for Improving Mental Healthcare Services

From the study findings, it was suggested that increasing outreach efforts and conducting awareness campaigns can improve mental healthcare services. According to Hampson, et al. [53], openly discussing mental health conditions can help reduce stigma and promote acceptance. Thus, by raising awareness, individuals experiencing mental health challenges can feel supported and encouraged to seek help. This may be achieved through mental health awareness campaigns that are designed to reach diverse populations, including vulnerable communities and remote areas. According to Gopalkrishnan and Babacan [54], ethno-specific approaches to mental health that incorporate traditional and community-based systems can provide new avenues for working with culturally diverse populations. As such, healthcare providers, community leaders, and mental health organizations can collaborate to design culturally sensitive campaigns to address specific cultural beliefs and barriers to mental healthcare. Moreover, incorporating peer support and testimonies from individuals who have successfully managed mental health challenges can make awareness efforts more relatable and impactful [55].

## 5. Summary, Practical Implications, and Recommendations of the Study

The impact of the COVID-19 pandemic on mental healthcare provision is extensive and requires a comprehensive response. Integrating telehealth, expanding psychoeducation efforts, addressing vaccine hesitancy, and prioritizing mental health support for both patients and healthcare workers are crucial steps in ensuring effective mental healthcare provision during crises. The lessons learned from the COVID-19 pandemic underscore the importance of adaptability and innovation in mental healthcare provision. Strategies such as increased outreach, prioritizing patients’ care, and utilizing technology and NGO support should be incorporated into standard mental healthcare practices. In doing so, healthcare systems can better address challenges during emergencies, improve patient outcomes, and enhance mental health support in both normal and crisis situations. The positive changes observed during the pandemic in terms of increased mental health awareness among healthcare professionals and younger generations are promising. However, challenges remain in enhancing awareness and acceptance of mental health among the broader community, including the elderly and less educated individuals. Targeted mental health education programs, community-based initiatives, and cultural sensitivity are essential in promoting mental health acceptance and improving mental healthcare provision in diverse communities.

Overall, the findings of this study offer valuable insights into enhancing mental healthcare services and public health responses during the COVID-19 pandemic. Arguably, we proposed that by implementing outreach and awareness campaigns, mental health services can become more accessible and accepted. Additionally, providing accurate information about COVID-19 and vaccinations is crucial to building trust and encouraging vaccine acceptance. In summary, this study conclusively suggests that by adopting the strategies highlighted, healthcare systems can promote better mental health outcomes and contribute to successful pandemic management. This is vital as the world prepares for another kind of pandemic in the near future. 

## 6. Limitations of the Study

With a small sample size, the findings of the study may not be easily generalized to a larger population of PHC nurses in Durban or beyond. The experiences and perspectives of the selected participants might not fully represent the diversity of views and challenges in the entire nursing community. For example, the variation in working years of professional experience, gender, diversified specialties, and the distribution of participants coming from private and public healthcare sectors have not been explored in this study. Further investigations or follow-up studies will be conducted to gain more insights into the influence of these variables in understanding the experiences of PHC nurses on mental health during COVID-19. Furthermore, the experiences and viewpoints of the selected nurses might not capture the full spectrum of mental health challenges faced in the region. This could lead to an incomplete understanding of the topic.

## 7. Conclusions

The impact of the COVID-19 pandemic on mental healthcare provision has been significant, bringing forth both challenges and positive changes. The challenges of reduced patient visits, the complexity of healthcare provision, and a lack of adequate support have underscored the need for adaptability and resilience in mental health services. On a positive note, increased awareness of mental health among healthcare professionals and younger generations has been observed during the pandemic. However, there is still work to be performed in raising awareness among the broader community, especially the elderly and less educated individuals.

To improve mental healthcare services, it is crucial to focus on outreach efforts, conduct awareness campaigns, and provide accurate information about mental health and COVID-19. Addressing stigma, promoting acceptance, and fostering a supportive environment are key to empowering individuals to seek help and support their mental well-being.

Additionally, cultural sensitivity and community engagement are essential to bridge gaps and address varying perceptions of mental health in diverse communities. Transparent communication about COVID-19 and vaccines is vital to building public trust and encouraging vaccine acceptance, thus contributing to better pandemic management.

## Figures and Tables

**Table 1:** Identification of themes and subthemes.

Themes	Subtheme
1. Impact of COVID-19 on Mental Healthcare Provision	Medication Adherence Challenges Fear and Uncertainty Reluctance to Vaccination Decrease in Clinic Visits Mental and Emotional Toll
2. Adaptation of Mental Healthcare Services	Increased Outreach and Support Prioritizing Patients’ Care Use of Technology and NGOs
3. Challenges in Providing Mental Healthcare Services	Reduced Number of Patients Complexity of Healthcare Provision during the Pandemic Lack of Adequate Support
4. Positive Changes from Providing Mental Healthcare Services during the pandemic	Awareness of Mental Health Community Acceptance of Mental Health
5. Suggestions for Improving Mental Healthcare Services	Outreach and Awareness Campaign Providing Accurate Information

## Data Availability

The data presented in this study are available upon request from the corresponding author. The data are not publicly available due to privacy restrictions.
